# Effects of Capital Flexion Exercise on Craniovertebral Angle, Trunk Control, Balance, and Gait in Stroke Patients with Forward Head Posture: A Randomized Controlled Trial

**DOI:** 10.3390/medicina61050797

**Published:** 2025-04-25

**Authors:** Dong-A Hyeon, Jeong-Seon Kim, Hyoung-Won Lim

**Affiliations:** 1 Department of Physical Therapy, Graduate School, Dankook University, Cheonan 31116, Republic of Korea; z_a63@naver.com; 2 Department of Physical Therapy, Gangdong University, Eumseong-gun 27690, Republic of Korea; 3 Department of Physical Therapy, College of Health Sciences, Dankook University, Cheonan 31116, Republic of Korea

**Keywords:** stroke, forward head posture, capital flexion exercise, postural control, gait

## Abstract

*Background and Objectives*: Forward head posture (FHP) is associated with reduced stability limits, impaired balance performance, and compromised cervical proprioception. This study investigated the effects of capital flexion exercise (CFE) on the craniovertebral angle (CVA), trunk control, balance, and gait in chronic stroke patients with forward head posture. *Materials and Methods*: Twenty-six subjects were randomly assigned to the CFE group or the control group (n = 13 each). The CFE group underwent a familiarization process and performed CFE for 9 min per session, 3 times a week for 6 weeks, as well as the existing neurodevelopmental treatment (NDT). The control group received only the existing NDT. *Results*: The CVA, the Korean version of the Postural Assessment Scale for Stroke (K-PASS), the Berg Balance Scale (BBS), and the Timed Up and Go test (TUG) improved after the intervention in the CFE group (*p *< 0.05). In the control group, CVA and TUG improved after the intervention (*p *< 0.05). The CVA (*d* = 1.34, *p* = 0.002), K-PASS (*d* = 1.36, *p* = 0.000), and BBS (*d* = 1.68, *p* = 0.000) values of the CFE group showed statistically significant improvement compared to the control group. Although TUG improved in the CFE group, the between-group difference was not statistically significant (*d* = −0.28, *p* = 0.467). *Conclusions*: This study suggests that capital flexion exercises effectively improve craniovertebral angle, trunk control, and balance in chronic stroke patients with forward head posture.

## 1. Introduction

Forward head posture (FHP) is the most common postural defect in the sagittal plane of the cervical spine. It is defined as a posture in which the head is placed in a forward direction, with excessive extension of the cervical vertebrae [1]. This is related to excessive extension of the upper cervical vertebrae (C1–C3) and flexion of the lower cervical vertebrae (C4–C7) [2]. The FHP causes many problems, such as neck pain [3], cervicogenic dizziness [4], limited transmission of proprioception [5], and load on the cervical vertebrae [6]. Kang et al. reported that adults aged 30 to 40 who had used a computer for more than 6 h at a time for 10 years exhibited a forward-shifted center of gravity and poorer balance than the control group [7]. Another study reported that people with chronic degenerative cervical myelopathy, in whom the center of gravity shifted forward, had impaired gait due to a decrease in proprioception when standing on one leg or supported by both feet [8].

Common disabilities in stroke patients include motor and sensory impairment, ab-normal balance ability, difficulty in shifting weight to the affected side, dysphagia, and cognitive impairment [9]. In particular, stroke patients lack dynamic postural control and are at a higher risk of falling than healthy individuals [10]. Damage to the vestibular system leads to compensatory strategies for gaze stabilization, with reduced amplitude and velocity of head movements [11]. Hemiplegic patients often show altered postural alignment, and FHP has also been observed in this population [12]. Given these challenges, FHP in stroke patients may further disrupt sensorimotor integration, limit cervical proprioception, and exacerbate balance deficits due to already compromised neuromuscular control. These compounded effects are likely to negatively influence functional recovery and increase the risk of falls during rehabilitation.

Capital flexion exercise (CFE) is an exercise that activates the deep neck flexor muscles, which are the deep muscles of the cervical vertebrae [13]. CFE straightens the cervical vertebrae, connects them to the thoracic vertebrae, lowers the ribcage, and tilts the pelvis posteriorly, resulting in movement of the entire spine [13]. This postural alignment enhances vestibular input and midline orientation, thereby improving postural tone and providing a foundation for stable balance and movement against gravity [13]. In a previous study, when school teachers with neck pain engaged in deep cervical flexor muscle training, significant improvements in pain and FHP were reported compared to the conventional exercise (stretching and strengthening) [14]. In another study, craniocervical flexor training intervention and endurance-strength training intervention were applied to patients with neck pain, and it was reported that the intervention was effective in terms of changing the craniocervical angle and reducing neck pain [15]. In a study of stroke patients, joint mobilization of the cervical vertebrae was applied to investigate the effects on the craniovertebral angle, respiratory function, and swallowing function [16,17]. However, to the best of our knowledge, no study has investigated the effects of CFE in stroke patients on the craniovertebral angle, postural control, and functional movement.

Therefore, this study aimed to investigate the effect of CFE on the craniovertebral angle (CVA), trunk control, balance, and gait in chronic stroke patients with FHP.

## 2. Materials and Methods

### 2.1. Participants

**Figure 1:**
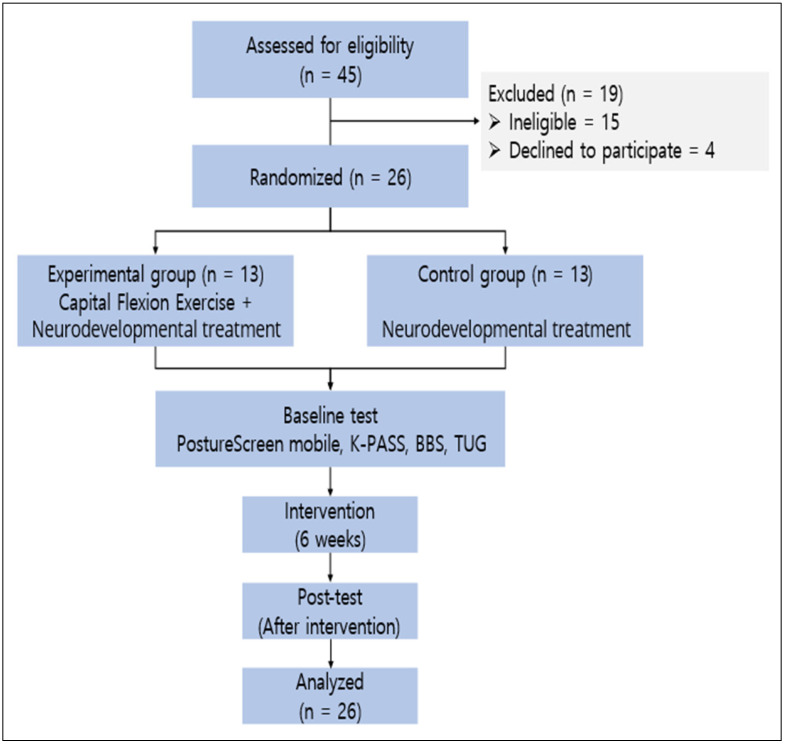
Flow of participants through the study.

This study was conducted in collaboration with two rehabilitation hospitals in Cheonan, South Korea. A total of 26 patients provided written informed consent and were enrolled—21 from Rehabilitation Hospital A and 5 from Rehabilitation Hospital B. Participants were then randomly assigned to either the experimental group (n = 13) or the control group (n = 13) by a blinded, independent research assistant using a computer-generated randomization sequence. The experimental group received both capital flexion exercise (CFE) and conventional neurodevelopmental treatment (NDT), while the control group received NDT only. In both groups, chronic stroke patients were evaluated before and after the 6-week intervention. The inclusion criteria were as follows: forward head posture (craniovertebral angle < 48°) [18], a minimum of 6 months post-stroke, ability to communicate (Mini-Mental State Examination-Korean version score of 24 or higher), ability to walk independently for 10 m without assistive devices, and no traumatic neck pain, hearing, or vision impairment. Unilateral spatial neglect was assessed using the Line Bisection Test and clinical observation, which are commonly used to detect visual-spatial inattention in stroke patients [19]. Participants showing signs of neglect were excluded. All participants adhered to the predefined protocol, with no significant deviations or dropouts. The intervention was consistently applied across both groups, and data collection followed the study’s management plan, ensuring the integrity and reliability of the results (Figure 1).

All participants provided written informed consent after receiving a detailed explanation of the study’s purpose and procedures. This study was approved by the Dankook University Institutional Review Board (approval number: 2021-03-057-001) and registered with the Clinical Research Information Service (KCT0008152). The participants confirmed their understanding of the study’s objectives and procedures. The study adhered to the ethical principles outlined in the Declaration of Helsinki.

### 2.2. Intervention

The exercise program in the experimental group consisted of conventional neurodevelopmental treatment (NDT), identical to that received by the control group, with the addition of capital flexion exercise (CFE). Sessions were conducted for 30 min, three times a week, over a six-week period. The NDT component included trunk control training, weight shifting, balance tasks, and functional mobility exercises. Trunk control training involved anterior-posterior and lateral pelvic tilts in a seated position, as well as trunk elongation in side-sitting. Weight-shifting exercises included therapist-guided lateral and diagonal shifts in a standing position. Balance tasks comprised static standing with reduced upper limb support and dynamic activities such as stepping and reaching in multiple directions. Functional mobility exercises included guided sit-to-stand transitions, gait training with verbal and tactile cues, and task-specific transfers. All exercises were tailored to each participant’s ability and progressively increased in complexity over the six-week period (e.g., transitioning from supported sitting to unsupported standing tasks). Prior to introducing CFE, a familiarization process was conducted to reduce neck tension. Stretching exercises, based on previous studies, were performed in a standing position, with the neck flexors, extensors, lateral flexors, and rotators each held for 30 s and repeated three times [20,21].

**Figure 2:**
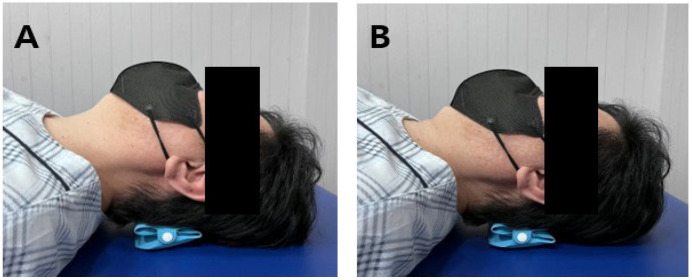
Capital flexion exercise using a pressure biofeedback device. (**A**) Starting position; (**B**) Capital flexion exercise.

During the exercise, participants were positioned with their foreheads and chins in horizontal alignment, knees bent at 90°, and a pressure biofeedback device (Stabilizer Pressure Biofeedback, Chattanooga, DJO, LLC, Carlsbad, CA, USA) placed below the occipital region. The device’s manometer was initially set to 20 mmHg relaxed state, and participants were instructed to nod their heads, gradually increasing pressure by 2 mmHg increments until reaching 30 mmHg [22]. The device has demonstrated good repeatability (ICC = 0.831), moderate intra-rater reproducibility (ICC = 0.685), and fair inter-rater reproducibility (ICC = 0.442) when used to assess deep neck flexor performance, supporting its reliability in both clinical and research settings [23]. During training, participants maintained a flexed neck position by drawing the chin in-ward to facilitate posterior neck muscle stretching. This posture was held for 10 s and repeated 10 times per set, with 3 sets totaling 9 min and two-minute rest intervals between sets (Figure 2) [15,24,25].

In contrast, the control group received conventional neurodevelopmental treatment, which included mat exercises and gait training. This was performed for 30 min, 3 times a week, over the same 6-week period. Both groups, consisting of chronic stroke patients with forward head posture (FHP), received an equal total treatment time of 30 min during the 6-week intervention. Pre-intervention and post-intervention evaluations were conducted for all participants.

### 2.3. Outcome Measures

To ensure consistency, a single blinded rater conducted all outcome assessments before randomization and after the 6-week intervention across both rehabilitation hospitals. The primary outcome measure was the change in craniovertebral angle (CVA) following CFE. Secondary outcomes included the effects of CFE on trunk control, balance, and gait in stroke patients with forward head posture. A single physical therapist evaluated the CVA, trunk control, balance, and gait of all patients both before and after the intervention. The CVA was measured using the Posture Screen Mobile (PSM) application. Trunk control was assessed using the Korean version of the Postural Assessment Scale for Stroke (K-PASS), and dynamic balance and gait were measured using the Berg Balance Scale (BBS) and Timed Up and Go test (TUG), respectively.

#### 2.3.1. Primary Outcome

Craniovertebral Angle (CVA): One examiner measured the CVA using PSM version 11.10 to assess forward head posture (FHP). The PSM application’s intra-class correlation coefficient (ICC) for anterior/posterior head shift has been reported to be 0.724, with reliability varying between ICC = 0.26–0.93 (inter-measurer) and ICC = 0.33–0.86 (intra-measurer) [26]. Nevertheless, a previous study reported that a similar tool to ours—Posture Pro 8 software—can be considered a reliable method for assessing various posture-related measures from photographs, particularly when used by the same examiner [27]. We selected the PSM application due to its accessibility, prior use in clinical research, convenience, and non-invasive nature. Participants were photographed in a standing position using an iPhone 12 mini (iOS 15.6) mounted on a tripod. The application provided measurements from the anterior, posterior, and lateral planes, with frontal plane landmarks displayed. A smaller CVA corresponds to more pronounced FHP, and a CVA of <48° was considered indicative of FHP, as referenced in previous studies [18,28].

#### 2.3.2. Secondary Outcome

Korean Version of Postural Assessment Scale for Stroke (K-PASS): The K-PASS is composed of 12 items—5 for posture maintenance and 7 for posture changes—scored on a scale from 0 to 3. A score of 0 indicates an inability to perform the task, while a score of 3 signifies the ability to perform it independently. The K-PASS demonstrates high reliability, with inter-rater reliability ranging from 0.88 to 0.98 and intra-rater reliability from 0.77 to 0.99 [29].

Berg Balance Scale (BBS): The BBS consists of 14 items, each scored from 0 to 4, for a maximum possible score of 56. These items are divided into three categories: sitting, standing, and posture change. Scores can be interpreted as follows: high fall risk (0–20 points), medium fall risk (21–40 points), and low fall risk (41–56 points). The BBS showed high intra-rater reliability (ICC = 0.99) and inter-rater reliability (ICC = 0.98) [30].

Timed Up and Go Test (TUG): The TUG test involved measuring the time taken for a participant to rise from a chair, walk 3 m, turn, return, and sit down. The intra-rater reliability for the TUG was ICC = 0.944–0.987, and the inter-rater reliability was ICC = 0.954–0.998 [30]. In this study, the average value of three repeated measurements was used.

### 2.4. Statistical Analysis

The general characteristics of the study participants were presented as the mean and standard deviation, serving as descriptive statistics. Due to the small sample size, normality of the population could not be assumed, and as a result, all analyses were conducted using non-parametric tests. The Wilcoxon signed-rank test was performed to examine the changes in craniovertebral angle (CVA), Postural Assessment Scale for Stroke (K-PASS), Berg Balance Scale (BBS), and Timed Up and Go test (TUG) within both the capital flexion exercise (CFE) group and the control group. Additionally, the Mann–Whitney U test was used to assess the differences in the amount of change in CVA, K-PASS, BBS, and TUG after the intervention between the CFE group and the control group. The data were statistically processed using SPSS for Windows version 21.0, and the statistical significance level was set at 0.05.

## 3. Results

### 3.1. General Characteristics of Participants

The general characteristics of the participants are presented in Table 1. The CFE group and the control group were found to be homogeneous in terms of their general characteristics (*p *> 0.05).

### 3.2. Comparison of Craniovertebral Angle Before and After the Intervention (Primary Outcome)

The CVA significantly increased in both groups after the intervention. In the CFE group, it improved from 45.12 ± 2.73° to 55.81 ± 4.43° (*p *< 0.05), while in the control group, it increased from 46.44 ± 1.37° to 50.40 ± 4.58° (*p *< 0.05). The improvement was significantly greater in the CFE group than in the control group (*p *< 0.01) (Table 2).

### 3.3. Comparison of Postural Assessment Scale for Stroke, Berg Balance Scale, and Timed up and Go Test Before and After the Intervention (Secondary Outcomes)

The K-PASS score increased significantly in the CFE group (32.46 ± 1.39 to 33.54 ± 1.61, *p *< 0.05), while the control group showed a slight decrease (32.15 ± 1.86 to 31.92 ± 1.93), which was not significant. The improvement was significantly greater in the CFE group (*p *< 0.01).

Similarly, the BBS score increased significantly in the CFE group (45.85 ± 5.83 to 49.54 ± 4.46, *p *< 0.05), whereas the control group showed a non-significant increase (41.00 ± 6.12 to 41.54 ± 5.99). The CFE group demonstrated significantly greater improvement (*p *< 0.01).

For the TUG test, both groups showed a significant decrease in completion time (CFE: 20.09 ± 13.33 to 17.85 ± 12.82 s, *p *< 0.05; Control: 29.29 ± 22.95 to 25.11 ± 15.42 s, *p *< 0.05). However, the between-group difference in improvement was not statistically significant (*p *> 0.05) (Table 2).

## 4. Discussion

This study explored the effects of capital flexion exercises (CFEs) on craniovertebral angle (CVA), trunk control, balance, and gait in chronic stroke patients with forward head posture (FHP). Significant improvements were observed in CVA, trunk control, and balance in the CFE group. However, no significant differences were found in gait between the groups. These findings suggest that CFE could be a valuable addition to rehabilitation programs for stroke patients with postural abnormalities, particularly for improving postural control and balance.

The observed improvements in CVA, trunk control, and balance are consistent with previous research on cervical stabilization exercises. Previous research demonstrates that exercises targeting cervical alignment, such as CFE, can effectively correct FHP and improve associated outcomes like neck pain and respiratory function [17,31]. Active cervical contraction, as opposed to passive techniques, may explain the superior postural correction observed, supporting findings that active cervical engagement enhances broader postural stability. These effects may be particularly relevant for stroke patients with FHP, where impaired postural alignment can exacerbate balance deficits.

The improvements in trunk control and balance observed in this study further support the notion that FHP contributes to compromised postural stability. Previous research has demonstrated that reduced craniovertebral angle (CVA) is associated with impaired balance [32,33], and CFE likely improves balance by enhancing proprioceptive feedback and engaging the vestibular system through active cervical flexion. This mechanism aligns with evidence showing that cervical stabilization enhances dynamic postural control by improving trunk muscle activity [34,35,36]. Furthermore, impaired cervical spine proprioception, often linked to muscle spindle dysfunction, is known to affect postural control.

The neck muscles—particularly the rectus capitis posterior, obliquus capitis, and longissimus capitis—contain abundant muscle spindles that play a critical role in maintaining postural stability [37,38]. In FHP, the shortening of occipital extensors and the lengthening of cervical extensors disrupt normal proprioceptive input, further compromising postural control [39,40]. CFE is thought to contribute to postural improvements by reducing the shortening of the occipital extensors and preventing the excessive lengthening of the cervical extensors. These changes likely help restore normal proprioceptive input from muscle spindles, improving the interaction between proprioceptive and vestibular systems and supporting better postural regulation. The interaction between proprioceptive and vestibular inputs further highlights the importance of cervical spine alignment in maintaining balance.

Although both groups showed significant within-group improvements in the Timed Up and Go (TUG) test, the between-group difference was not statistically significant. This may be attributed to the multifactorial nature of TUG performance, which requires coordinated integration of balance, postural control, lower extremity strength, proprioception, and gait speed [41]. While the capital flexion exercise (CFE) targeted upper cervical control and postural alignment, it may have had limited direct influence on lower limb function and overall gait performance. Furthermore, both groups received conventional neurodevelopmental treatment (NDT), which likely contributed similarly to improvements in functional mobility. Although this explanation is supported by previous literature, we acknowledge that it remains speculative, as no subgroup analyses were conducted to directly investigate the relationship between postural control and gait. Future studies are needed to explore these associations and better understand the pathways through which postural interventions may influence gait-related outcomes.

In summary, this study highlights the potential value of incorporating CFE into neuro-developmental treatment to enhance postural control in stroke patients with FHP. While the specific clinical implications of these improvements require further investigation, our findings suggest that targeted cervical exercises may support balance rehabilitation in this population. Future research should further clarify the functional significance of these outcomes and address limitations such as the short intervention period and lack of long-term follow-up.

### Limitations

It is crucial to recognize several limitations that may affect the interpretation and generalizability of these results. First, the relatively small sample size limits the statistical power of the study, making it difficult to extend the findings to a broader stroke population. This limitation is common in clinical trials, particularly those focused on specific subgroups of stroke patients, and future studies should aim to recruit larger, more diverse cohorts. Additionally, the study was conducted in two rehabilitation hospitals in Cheonan, South Korea, which may not reflect the characteristics of stroke populations in other regions, thereby reducing the external validity of the findings. Another limitation is the relatively short intervention period and the lack of long-term follow-up. Although improvements in trunk control and balance were observed, it remains unclear whether these gains are sustainable over time. Finally, objective tools such as electrophysiological assessments, imaging, or center of gravity monitoring were not used, which may have provided more detailed insight into the neuromuscular and postural mechanisms underlying the effects of CFE. Future studies should consider incorporating these measures for a more comprehensive analysis.

Despite these limitations, the study has several methodological strengths that support the robustness of its findings. Randomization and allocation concealment were implemented to minimize bias, and assessors were blinded to the group allocation for primary outcomes. Moreover, data analysis followed the intention-to-treat principle, preserving the integrity of the randomization process and ensuring that all participants were included in the final analysis. These methodological features enhance the credibility and reliability of the study’s outcomes.

Clinically, CFE could be integrated into stroke rehabilitation programs as a targeted intervention for patients with FHP, focusing on postural control and balance. Based on the improvements observed in this study, therapists might consider incorporating CFE into neurodevelopmental or balance-focused sessions, with frequency adjusted to individual needs (e.g., 3–5 sessions per week). Combining CFE with other interventions, such as lower-limb strengthening or gait-specific exercises, may further enhance functional outcomes for stroke patients.

## 5. Conclusions

This study demonstrates that capital flexion exercises (CFEs) improve forward head posture (FHP), trunk control, and balance in chronic stroke patients with postural abnormalities. These findings suggest that CFE could be an effective intervention for addressing postural control deficits in stroke rehabilitation. Future studies should focus on larger sample sizes, extended follow-up periods, and diverse clinical settings to further establish the long-term efficacy of CFE, particularly in combination with other rehabilitation interventions aimed at improving gait and functional mobility.

## Figures and Tables

**Table 1:** Baseline characteristics of study participants.

Variables	Groups	
	CFE (n = 13)	Control (n = 13)
Age (year), mean (SD)	55.38 (8.60)	58.69 (9.12)
Height (cm), mean (SD)	166.31 (9.79)	167.85 (7.09)
Weight (kg), mean (SD)	64.00 (15.18)	64.38 (10.46)
Gender, n male (%)	8 (62.0)	11 (85.0)
K-MMSE (score), mean (SD)	26.46 (2.50)	27.00 (2.12)

CFE: Capital flexion exercise; K-MMSE: Mini-Mental State Examination-Korean version.

**Table 2:** Mean (SD) values, within-group and between-group differences (95% CI) for craniovertebral angle, postural assessment scale for stroke, Berg balance scale, and timed up and go test.

Outcome	Groups	Baseline	Post-Intervention	*p*-Value (Within Group)	*p*-Value (Between Groups)	Effect Size (*d*)
CVA (degree)	CFE (n = 13)	45.12 ± 2.73	55.81 ± 4.43	0.001	0.002	1.34
	Control (n = 13)	46.44 ± 1.37	55.40 ± 4.58	0.023		
PASS (points)	CFE (n = 13)	32.46 ± 1.39	33.54 ± 1.61	0.002	0.000	1.36
	Control (n = 13)	32.15 ± 1.86	31.92 ± 1.93	0.083		
BBS (points)	CFE (n = 13)	45.85 ± 5.83	49.54 ± 4.46	0.001	0.000	1.68
	Control (n = 13)	41.00 ± 6.12	41.54 ± 5.99	0.248		
TUG (seconds)	CFE (n = 13)	20.09 ± 13.33	17.85 ± 12.82	0.006	0.467	−0.28
	Control (n = 13)	29.29 ± 22.95	25.11 ± 15.42	0.039		

Mean ± SD; CFE: Capital flexion exercise; CVA: Craniovertebral angle; PASS: Postural assessment scale for stroke; BBS: Berg balance scale; TUG: Timed up and go test.

## Data Availability

Original data are available from the corresponding author upon reasonable request.
